# Not too rigid nor too wobbly: Defining an optimal membrane fluidity range essential for biofilm formation in *Escherichia coli*

**DOI:** 10.1002/mlf2.70024

**Published:** 2025-08-04

**Authors:** Yaoqin Hong, Jilong Qin, Makrina Totsika

**Affiliations:** ^1^ Centre for Immunology and Infection Control, School of Biomedical Sciences Queensland University of Technology Herston Queensland Australia; ^2^ Max Planck Queensland Centre Queensland University of Technology Herston Queensland Australia; ^3^ Biomedical Sciences and Molecular Biology, College of Medicine and Dentistry James Cook University Douglas Queensland Australia; ^4^ Institute for Molecular Bioscience University of Queensland St Lucia Queensland Australia

## Abstract

Membrane fluidity plays a crucial role in bacterial fitness and adaptation to cope with rapid environmental changes. While high membrane fluidity promotes robust biofilm formation in *Klebsiella pneumoniae*, studies in several other species, including *Salmonella enterica*, suggest that biofilm formation is associated with reduced fluidity. This paradox may reflect the complex relationship between lipid composition and biofilm formation. Our findings demonstrated that both low and high extremes of lipid fluidity restrict biofilm formation. We propose that the required fluidity for biofilm growth, relative to that required for planktonic growth, may differ between species and is readily adjusted to fall within a “Goldilocks” range during lifestyle transitions.

Microbes in natural environments often exist in communities, called biofilms, where cells are encased in an extracellular polymeric matrix (EPS) that provides protection from external stresses such as detergents and antibiotics. This EPS is primarily composed of polysaccharides, proteins, lipids, and extracellular DNA, with its composition and spatial organization tightly regulated by molecular signals^1^. Identifying the molecular cues that bacteria use to regulate biofilm formation and dispersal is critical for both combating infections and harnessing beneficial biofilms. The contribution of bacterial cell membrane fluidity, as a modulator of these processes, remains an underexplored area.

The bacterial membrane plays a vital role in supporting essential processes, including energy generation, fitness in different environments, and changes in environmental conditions that necessitate membrane lipid adjustments for survival and fitness^2^. Changes in membrane lipids serve as a molecular cue in the Envelope Stress Response that can influence various pathways involved in biofilm development. A typical bacterial phospholipid contains two fatty acid (FA) chains, and the chemical nature of these FAs dictates the membrane fluidity^2^. Previous studies have demonstrated that biofilms of *Salmonella enterica, Staphylococcus aureus, Listeria monocytogenes*, and *Pseudomonas aeruginosa* contain elevated levels of saturated fatty acids (SFAs) compared to their planktonic counterparts^3^. This suggests that bacterial membrane composition and fluidity may play roles in biofilm formation, although the underlying mechanisms remain unclear^3^, ^4^.

Most bacterial species de novo synthesize the acyl chains to be integrated into membrane lipids by the highly conserved type II FA synthesis pathway distinctive from that used by eukaryotes^2^. As such, the pathway had served as an important target for antimicrobial discovery^5^, ^6^, with the recent progress showing the potential of potentiating susceptibility to otherwise ineffective antibiotics^7^, ^8^. In *Escherichia coli*, the major unsaturated fatty acids (UFAs) are palmitoleate (C16:1) and cis‐vaccenate (C18:1). Bacterial phospholipids show chemical asymmetry, with SFAs typically occupying the *sn*1 position and UFAs occupying the *sn*2 position of the glycerol backbone due to the substrate preference of acyltransferases involved in phosphatidic acid synthesis^2^, ^9^. The UFA/SFA ratio that affects membrane fluidity is thus predominantly determined by the diversity of FAs at the *sn*2 position^2^, ^9^. The only exception to this substrate selectivity at position *sn*1 is the integration of cis‐vaccenate, which leads to higher membrane fluidity that is imperative for bacterial fitness under certain conditions, such as low‐temperature growth^10^. As such, this phospholipid asymmetry plays a pivotal role in modulating membrane fluidity, which, in turn, may influence biofilm formation. In *E. coli*, UFA synthesis is mediated by ketoacyl‐ACP synthase I (FabB), while the conversion of palmitoleate into cis‐vaccenate requires ketoacyl‐ACP synthase II (FabF)^11^, ^12^. Thus, FabB and, to a lesser extent, FabF, are key players governing membrane fluidity. Interestingly, while FabB catalyzes the rate‐limiting step in UFA synthesis and governs the fundamental basis of membrane fluidity, the enzyme is not thermoregulated^13^. Rather, the FabF enzyme involved in the final conversion from palmitoleate into cis‐vaccenate was reported to be under thermal regulation across phyla, including the *Firmicutes* ^14^. The two enzymes function in concert to regulate membrane fluidity and optimize fitness required for survival across a wide range of temperatures.

The *E. coli* K‐12 strain AR3110 shows a distinct macrocolony architecture when grown on agar surfaces (Figure 1A), characterized by a radial, rugose, three‐dimensional structure with delaminated buckling and skin‐like elasticity^15^. This strain also forms a robust pellicle highly resistant to mechanical disturbance at the liquid–air interface (Figure 1B). Remarkably, both the rugose colony architecture and pellicle formation disappeared in the ∆*fabF* strain, suggesting that reduced membrane fluidity underlies these phenotypic changes (Figure 1A,B).

**Figure 1:**
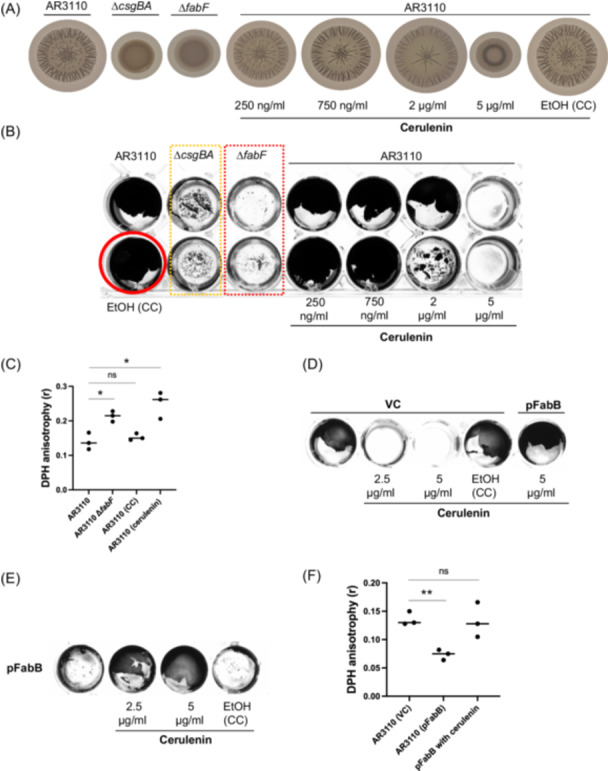
Optimal membrane fluidity governs *Escherichia coli* biofilm rugosity and pellicle formation. (A) The *fabF* null mutation or cerulenin treatment reduced rugosity in AR3110 macrocolony biofilms grown on YESCA agar at 26°C. (B) The *fabF* null mutation or cerulenin treatment inhibited pellicle formation in AR3110 cultured in Mueller–Hinton broth. Identical results were observed on YESCA, LB Lennox, and LBON. (C) 1,6‐Diphenyl‐1,3,5‐hexatriene (DPH) fluorescence anisotropy was used to measure membrane fluidity (note: fluidity is inversely proportional to fluorescence anisotropy). (D) Ectopic expression *of fabB* (pSU2718 vector, induced with 250 μM IPTG) restored pellicle formation inhibited by cerulenin treatment. (E) Overexpression of *fabB* blocked pellicle formation in AR3110, but this defect was alleviated by cerulenin treatment. (F) Ectopic expression of *fabB* significantly increased membrane fluidity, and this increase was ameliorated by 5 μg/ml cerulenin treatment. The AR3110 ∆*csgBA* strain used in panel (A) is unable to synthesize curli^15^, an essential structural component of AR3110 rugose macrocolonies. Panels (A), (B), (D), and (E) are representative of at least three independent observations. Data in panels (C) and (F) were obtained from three independent repeats. Error bars indicate the standard deviation. Statistical significance was determined using an unpaired, two‐tailed Student's *t*‐test. ns, not significant (*p* ≥  0.05); *p <  0.05; **p <  0.01. CC, carrier control (EtOH, ethanol used to dissolve cerulenin); VC, vector control (pSU2718).

Aside from the SFA/UFA ratio, membrane fluidity is also influenced by the profile of phospholipid headgroups. For example, phosphatidylethanolamine has a smaller headgroup than phosphatidylcholine, in this case allowing the formation of hydrogen bonds between cationic amino headgroups and the anionic phosphate residue of nearby lipids to affect membrane packing^16^, ^17^. Consistent with this, a reduction of membrane phosphatidylethanolamine content was observed for yeast cells at higher temperature growth^18^. Weibel and colleagues also demonstrated that cardiolipin deficiency results in the reduction of biofilm formation in *E. coli* ^4^. In this study, rather than using standard FA profiling, we utilized 1,6‐Diphenyl‐1,3,5‐hexatriene (DPH) fluorescence anisotropy, a common method for measuring membrane fluidity. Reduced anisotropy correlates with higher membrane fluidity, while higher anisotropy indicates a more ordered and less fluid membrane. The measured anisotropy in the ∆*fabF* strain was significantly higher compared to the wild‐type AR3110 control (*t‐*test, *p* = 0.0111) (Figure 1C), which supported the hypothesis that membrane fluidity is crucial for biofilm formation in *E. coli* (Figure 1C).

Cerulenin inhibits both FabF and FabB, enzymes crucial for the synthesis of UFAs, thereby reducing membrane fluidity^19^. To investigate the role of membrane fluidity in biofilm formation, AR3110 was treated with subinhibitory concentrations of cerulenin in both rugose and pellicle biofilm models (Figure S1). This treatment reduced membrane fluidity (*t‐*test, *p* = 0.0144) to levels similar to those observed in the ∆*fabF* strain (Figure 1C). The macrocolony of AR3110 typically shows a skin‐like consistency, which prevents full resuspension of the biomass in solution (Figure S2). However, both the ∆*fabF* strain and cerulenin‐treated wild‐type macrocolonies acquired a buttery consistency, allowing for easy resuspension (Figure S2). The data shown in Figure 1A,B demonstrated the absence of rugose macrocolony architecture and pellicle formation in both the ∆*fabF* strain and 5 μg/ml cerulenin‐treated wild‐type AR3110, suggesting that reduced membrane fluidity hinders the development of stable biofilm structures.

The ectopic expression of *fabB* counteracted the fluidity reduction of AR3110 caused by cerulenin treatment (*t‐*test, *p* = 0.8829) (Figure 1F), thereby restoring pellicle formation in cerulenin‐treated AR3110 (Figure 1D). This observation suggests that a reduction in membrane fluidity antagonizes biofilm development. While this manuscript was being prepared, a similar observation was reported in *Klebsiella pneumoniae* ^20^. FabR negatively regulates the expression of *fabA* and *fabB* in known *Enterobacteriaceae* species, including *K. pneumoniae*. Consequently, the activity of UFA synthesis in the *K. pneumoniae* ∆*fabR* mutant is elevated^20^, leading to higher membrane fluidity compared to the WT during biofilm growth. In agreement with our observation, the *K. pneumoniae* ∆*fabR* mutant was found to produce robust biofilm capable of withstanding mechanical disruptions^20^. Dramé et al.^20^ and this study collectively suggest that high membrane fluidity is beneficial for the robust development of microbial biofilms.

An intriguing aspect of biofilm lipid composition is the observed higher SFA content in biofilms compared to planktonic cells in several species, including *S. enterica* ^3^. This observation seems to contradict the idea that increased membrane fluidity is essential for biofilm formation. However, we hypothesize that this paradox arises from the complex relationship between lipid composition and biofilm formation. While membrane fluidity is crucial, an overabundance of UFAs may lead to excessive fluidity, compromising biofilm robustness.

In this study, we ectopically overexpressed *fabB* to enhance cis‐vaccenic acid production and suppress the biofilm‐inhibitory activity of cerulenin^13^ (Figure 1D). Strikingly, in the absence of cerulenin treatment, AR3110 cells ectopically expressing *fabB* showed significantly more fluid membrane (*t‐*test, *p* = 0.0021) (Figure 1F) and blocked pellicle formation in liquid culture (Figure 1E), supporting our hypothesis that an optimal balance of lipid fluidity, instead of simply high fluidity, is critical for biofilm formation.

To this end, we argue that the precise UFA/SFA ratio optimal for biofilm growth relative to that required for planktonic growth may vary across species if not at strain levels, and is readily adjusted to fall within an ideal “Goldilocks” zone and facilitate the transition into biofilm lifestyle^3^, ^4^, ^20^. Our findings highlight the critical role of optimized membrane fluidity for robust biofilm development, and these insights could pave the way for future studies exploring lipid composition as a potential target for biofilm control in clinical and industrial applications.

## AUTHOR CONTRIBUTIONS

**Yaoqin Hong**: Conceptualization; data curation; formal analysis; funding acquisition; investigation; methodology; project administration; resources; supervision; validation; writing—original draft; writing—review and editing. **Jilong Qin**: Resources. **Makrina Totsika**: Funding acquisition; project administration; supervision; writing—review and editing.

## ETHICS STATEMENT

This study did not involve any human participants or animal subjects.

## CONFLICT OF INTERESTS

M. T. is an employee of the GSK group of companies. All the remaining authors declare no competing interests. This study was conducted in the absence of any commercial or financial relationships that could be construed as a potential conflict of interests.

## Supporting information

SUPPLEMENTAL MATERIAL.

## Data Availability

All data generated or analyzed in this study are included in this article.
